# Identification of Markers on the Basis of Transcriptomic Analysis for Molecular Assignment of Medulloblastoma

**DOI:** 10.3390/ijms27135720

**Published:** 2026-06-24

**Authors:** Sergio Juárez-Méndez, Aarón Vázquez-Jiménez, Josselen Carina Ramírez-Chiquito, Vanessa Villegas-Ruíz, Ana Maria Niembro-Zuñiga, José Eduardo Farfán-Morales, Alfonso Marhx-Bracho, Edgar Krötzsch, Miguel Rodríguez-Morales, Emma Segura-Solís, Mario Perezpeña-Diazconti, Cecilia Ridaura-Sanz, Roberto Rivera-Luna, Pilar Eguía-Aguilar, Osbaldo Resendis-Antonio, Jorge Melendez-Zajgla

**Affiliations:** 1 Experimental Oncology Laboratory, National Institute of Pediatrics, Mexico City 04530, Mexicovanessavillegasruiz@yahoo.com.mx (V.V.-R.); 2 Molecular Pathology Laboratory, Department of Pathology, National Institute of Pediatrics, Mexico City 04530, Mexico; mpdiazconti@gmail.com; 3 Human Systems Biology Laboratory, National Institute of Genomic Medicine, Mexico City 14610, Mexico; avazquez@inmegen.gob.mx (A.V.-J.); oresendis@inmegen.gob.mx (O.R.-A.); 4 Postgraduate in Biological Sciences, Postgraduate Unit, Building D, 1st Floor, Postgraduate Circuit, University City, Coyoacán, Mexico City 04510, Mexico; 5 Department of Pediatric Oncology, National Institute of Pediatrics, Mexico City 04530, Mexico; 6 Department of Pathology, National Institute of Pediatrics, Mexico City 04530, Mexicoeseguras@pediatria.gob.mx (E.S.-S.);; 7 Department of Surgery, National Institute of Pediatrics, Mexico City 04530, Mexico; 8 Laboratory of Connective Tissue, National Institute of Rehabilitation “Luis Guillermo Ibarra Ibarra”, Mexico City 04530, Mexico; kroted@yahoo.com.mx; 9 Department of Human Genetics, National Institute of Pediatrics, Mexico City 04530, Mexico; 10 Medicine Faculty, National Autonomous University of Mexico, Mexico City 04510, Mexico; 11 Research Direction, National Institute of Pediatrics, Mexico City 04530, Mexico; riveraluna@yahoo.com; 12 Experimental Pathology Research Laboratory, Children’s Hospital of Mexico, Federico Gómez, Mexico City 06720, Mexico; eguiapilar@yahoo.com.mx; 13 Department of Clinical and Experimental Pathology, Children’s Hospital of Mexico, Federico Gómez, Mexico City 06720, Mexico; 14 Center for Complexity Sciences, National Autonomous University of Mexico, Mexico City 04510, Mexico; 15 Coordination of Scientific Research-Research Support Network, National Autonomous University of Mexico, Mexico City 14080, Mexico; 16 Functional Genomics Laboratory, National Institute of Genomic Medicine, Mexico City 14610, Mexico

**Keywords:** medulloblastoma, molecular assignment, gene expression, spatial transcriptome analysis

## Abstract

Medulloblastoma is a heterogeneous solid tumor, and its molecular characteristics are the most important prognostic factors for this neoplasm. Unfortunately, the molecular classification of MB-G3 and MB-G-4 medulloblastoma is very complex because of molecular similarity. Therefore, in this work, through unsupervised machine learning-based gene expression profiling, we identified a low molecular profile associated with four molecular groups of medulloblastoma. We performed medulloblastoma expression microarray data mining via the Partek Genomics Suite and Transcriptome Analysis Console (TAC), and we included a total of 25 fresh medulloblastoma tumors that were obtained and hybridized into HG U133 Plus 2.0 Array microarrays. To identify the molecular groups of the 25 patients, we compared them against classified patients, which were obtained from free repositories, and through data mining based on gene expression, compared the expression profiles of our patients. To do so, we performed an analysis via the least squares method via PCA. The molecular groups MB-WNT and MB-SHH were confirmed via immunohistochemistry via β-catenin, YAP1 and GAB1 antibodies in tissue fixed in formalin and embedded in paraffin, and another tissue section was placed on a Visium Spatial slide to perform spatial RNA-seq via Illumina NextSeq 2000 platform sequencers. The data obtained were analyzed with R. We identified the expression profiles associated with the four molecular groups and formed a reference set. Through unsupervised analysis via the least squares method, we assigned the molecular profiles of 25 patients with medulloblastoma, via the integration of bulk and spatial tumor molecular gene expression profiling analysis and with immunohistochemical findings, this strategy was fast and accurate. We observed correlations in three of the trials carried out and, in part, in one study, a patient who presented two tumor strains and two molecular signatures (SHH and G4), which led us to believe that this patient presented mixed phenotypic characteristics. Multigene expression profile analysis of medulloblastoma represents a significant advance in precision medicine; integrating different layers of transcriptomic information allows us to demonstrate underlying molecular changes in the four molecular groups that are essential for personalized therapy.

## 1. Introduction

Medulloblastoma (MB) is a priority health problem because it is the central nervous system (CNS) tumor with the highest incidence worldwide, with approximately 10.3 cases per million/year [1,2,3]. This neoplasm has a bimodal incidence; it is between 3–4 and 7–8 years of age and is the main cause of morbidity and mortality from CNS tumors in children [4].

MBs are embryonic tumors that originate in the posterior fossa of the cerebellum and present extensive intratumoral heterogeneity [5], reflecting a wide variety of tumor lines. Metastasis is very common at diagnosis (~30–40%), especially in infants [6,7], resulting in an unfavorable prognosis.

The degree of malignancy is variable, and several clinical factors are involved in MB prognosis, such as age (<3 years old), metastatic disease and degree of tumor resection. However, molecular classification, which includes four molecular groups, is the most important prognostic factor for survival [8]. The MB with the best prognosis is those in which the WNT pathway is activated (MB-WNT), accounting for approximately 10% of the cases with 5-year survival rates of up to 95% [9]. MBs that activate the SHH pathway (MB-SHH) account for approximately 30% of the cases. The survival of MB of SHH (MB-SHH) is dependent on genetic alterations, such as TP53 mutation, which is less than 50% [9,10], whereas the survival of the TP53 wild type is as high as 85% [9,10]. The MBs of Group 4 (MB-G4) are categorized as intermediate forecasts, as are the MB-SHHs with wild-type TP53 [9,10]. Additionally, age, advanced disease and metastasis at diagnosis must be considered for this molecular group. Finally, the MB of Group 3 (MB-G3) has the worst prognosis, and the risk increases when MYCN is amplified, resulting in very poor survival [11,12]. These are molecular characteristics are essential at diagnosis.

Current treatments for MB include surgery, radiation therapy, and chemotherapy. However, what has increased overall survival to >85% in developed countries has been therapy directed at the molecular group. With respect to MB-WNT, several clinical trials have focused on reducing therapy administration due to the favorable prognosis associated with this group and with the aim of reducing treatment-related toxicity and long-term adverse effects. For MB-SHH, the strategy is the administration of targeted therapy, including vismodegib [13,14], ribociclib, sonidegib, and prexasertib combined with chemotherapies [15]. Finally, for high-risk MB patients, given the poor response to treatment, clinical trials have focused on more intense treatments, which may include the administration of pemetrexed, gemcitabine, ribociclib and prexasertib [15].

Unfortunately, understanding the molecular groups of MB in our country has been among the greatest challenges. Therefore, it was not possible for our patients with MBs to receive more personalized treatments. Consequently, our patients are treated considering only the histopathological characteristics, which is why these patients were overtreated and undertreated; thus, our overall survival at 5 years was less than 50%. For this reason, the objective of this work was to identify potential molecular markers associated with four molecular groups of medulloblastoma and, on the basis of a multitranscriptomic strategy, to classify our patients, which will allow us to establish a framework for the personalized management of patients with medulloblastoma in our country.

## 2. Results

### 2.1. Expression Profile Associated with the Molecular Group of Medulloblastoma

**Figure 1:**
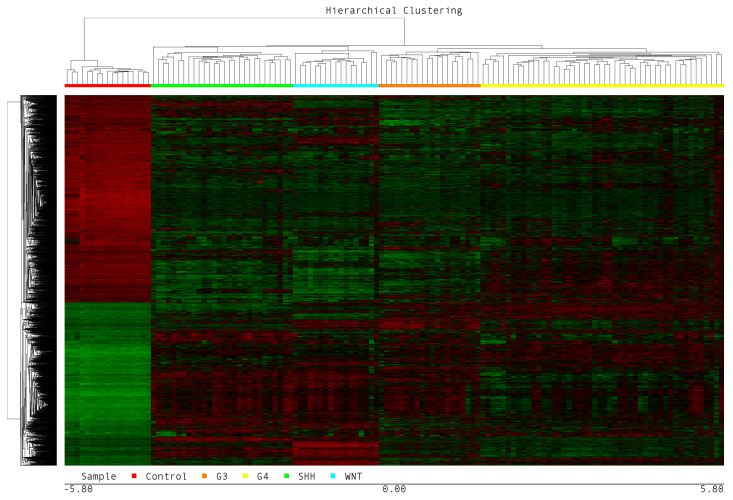
Expression profiles associated with the molecular subgroups of medulloblastoma. The figure shows the heatmap. In the upper part, the dendrogram of the grouped samples from left to right consists of a normal cerebellum (Control), *n* = 17 (red bar); MB-SHH, *n* = 28 (green bar); MB-WNT, *n* = 17 (blue bar); MB-G3, *n* = 20 (orange bar); and MB-G4, *n* = 48 (yellow bar). The dendrogram shows the differentially expressed genes in the four molecular groups of patients with medulloblastoma compared with the normal cerebellum.

Our microarray dataset (cell file) consisted of MB-WNT, MB-SHH MB-G3, MB-G4 and the normal cerebellum (control) (Supplementary Table S1). The GEP of the MB molecular group was obtained via comparative analysis in which normal cerebellum tissue was used for baseline expression, and the main comparison groups. This analysis allowed us to identify the expression profile associated with the four molecular groups, where MB-G3 presented a differential expression profile composed of 3338 DEGs, of which 1362 were overexpressed and 1976 were suppressed. In MB-G4, the GEP included 2705 DEGs, of which 1154 DEGs were overexpressed and 1551 were suppressed. In the MB-SHH group, 3355 DEGs were found; 1485 were upregulated, and 1870 were downregulated. Finally, in the MB-WNT group, 3967 DEGs were found; 1970 were overexpressed, and 1997 were suppressed. The data were obtained with an FDR value < 0.005 and a level of change of >5 and <−5 (fold change). The DEGs obtained are represented in the heatmap shown in Figure 1.

**Figure 2:**
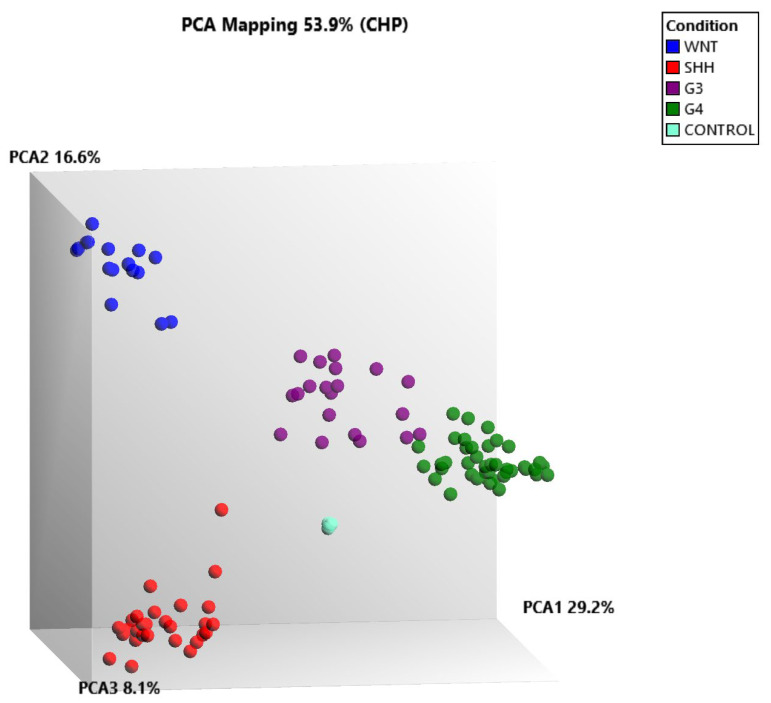
Molecular grouping of medulloblastoma via principal component analysis (PCA). The figure shows the MB-WNT (blue), MB-SHH (red), MB-G3 (purple), and MB-G4 (green) groups and the control group (sky blue). PCA clearly revealed the separation of the four molecular groups of MB. The reduction in the dimensional space could separate completely into MB-WNT, MB-SHH and the controls. Although MB-G3 and MB-G4 presented very similar expression profiles, in our analysis, we can differentiate them.

Since the initial transcriptomic analysis was performed using the commercial software Partek Genomics Suite v7.18, we subsequently sought to replicate the results employing a free-access tool, the Transcriptome Analysis Console (TAC) (Thermo Scientific, Waltham, MA, USA). This approach was chosen to validate the findings, reduce dependence on costly commercial licenses, and enable faster, more accessible transcriptomic analyses for future clinical cases. We transformed CEL files to CHPs in the TAC as follows: control *n* = 3, MB-WNT *n* = 17, MB-SHH *n* = 28, MB-G3 *n* = 20, and MB-G4 *n* = 42. Next, the categorization of the molecular groups was carried out via data mining, and as expected, through supervised grouping, it was possible to observe clear differences in the groupings of the four molecular groups (Figure 2). Despite the reduction in the size of our sample, we still have a consistent grouping between the four molecular groups of the MB and the controls as well as those observed via a heatmap (Figure 1).

### 2.2. The Reference Dataset Allows the Molecular Assignment of Patients with Medulloblastoma

**Figure 3:**
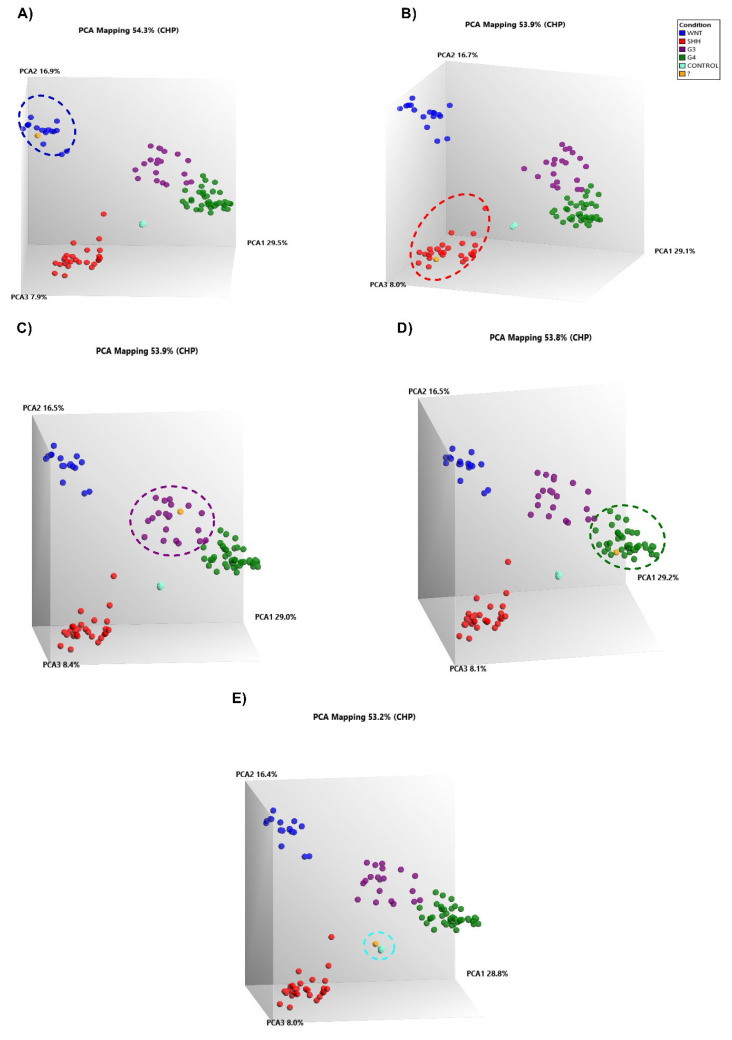
Prediction of the molecular groups of patients with medulloblastoma. The figure shows five PCAs, where the blue spheres represent MB-WNT, the red spheres represent MB-SHH, the purple spheres represent MB-G3, the green spheres represent MB-G4, the sky-blue spheres represent the controls, and the orange spheres represent unclassified MB patients. (**A**) The figure shows a representative sample of unclassified MB, which is grouped with the MB-WNTs. (**B**) The figure shows a representative sample with an unclassified MB, which is grouped with the MB-SHH. (**C**) The figure shows a representative sample with an unclassified MB, which is grouped with MB-G3. (**D**) The figure shows a representative sample with an unclassified MB, which is grouped with MB-G4. (**E**) The figure shows a representative sample of unclassified MBs, which is grouped with the controls.

Next, we identified the molecular groups of our MB patients via gene expression and data mining analyses. For this purpose, we applied the unsupervised analysis based on dimensional reduction using PCA via TAC software. In the analysis, we assigned our patients into corresponding molecular groups (Figure 3). All patients who did not have a molecular diagnosis were labeled as unknown (?) and with an orange color and were incorporated into the reference set in the unsupervised analysis. The results of our analysis are shown in Figure 3, where the principal component analysis (PCA) and clustering results of our cases have similar profiles within the corresponding molecular groups obtained via data mining. A representative patient with MB-WNT is shown in Figure 3A. One patient who had an MB-SHH is shown in Figure 3B. Patients grouped with MB-G3 are shown in Figure 3C. The representative grouping of one patient with MB-G4 is shown in Figure 3D. Finally, one patient whose sample was grouped with normal cerebellar tissue is shown in Figure 3E. We believe that this biopsy processed for the microarray analysis was mostly normal tissue, and for that reason, it was grouped with the controls. Unfortunately, we were unable to confirm this, owing to the limited size of the biopsy obtained.

### 2.3. Confirmation of the Molecular Assignment of MB-WNT and MB-SHH

**Figure 4:**
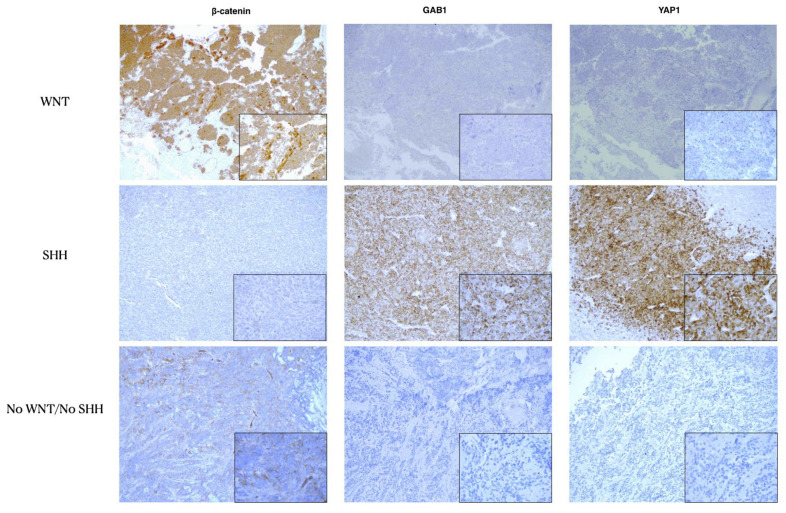
Confirmation of the molecular groups of the MB WNT and SHH genes. The figure shows the immunopositivity of β-catenin, YAP1 and GAB1. The upper row shows an MB-WNT, where the expression of nuclear β-catenin is observed in ~10% of the tumor cells and is negative for YAP1 and GAB1. In the middle row, an MB-SHH is shown, where the expression of YAP1 and GAB1 is observed and β-catenin is not expressed. The bottom row shows the negative expression of nuclear β-catenin, YAP1 and GAB1, suggesting that this patient was MB-G3 or MB-G4.

A total of 25 biopsies from patients diagnosed with medulloblastoma at the National Institute of Pediatrics and Children’s Hospital of Mexico, Federico Gómez, were evaluated via microarray gene expression, the results of which suggested that *n* = 4 were MB-WNT, *n* = 10 were MB-SHH, *n* = 6 were MB-G3, *n* = 4 were MB-G4, and one sample that clustered with the controls. After that, we proceeded to corroborate these results, at least for the molecular groups WNT and SHH, since it is clearly reported that the translocation of β-catenin to the nucleus confers the identity of MB-WNT and that nuclear/cytoplasmic YAP1 and cytoplasmic GAB1 confer identity for MB-SHH [16]. As expected, our immunohistochemistry results were in agreement with those of the microarray analysis; we observed nuclear positivity of β-catenin in the four cases that were grouped with the MB-WNT. Similarly, we observed the positivity of YAP1 and/or GAB1 in the nine cases that were identified by microarrays as MB-SHHs and included the case that was grouped with the controls. We also detected negative results for the previous marker in the remaining ten patients, suggesting that these patients were MB-G3 or MB-G4 (no-WNT/no SHH) (Figure 4).

### 2.4. Spatial Transcriptomics of Medulloblastoma Confirms the Assignment of Molecular Groups

We performed molecular assignment of our patients with medulloblastoma on the basis of GEP via a reference set and spatial transcriptome analysis (STA), which could be an alternative tool to determine the molecular diagnosis of medulloblastoma. We included five patients with STA, and in two patients, we analyzed two tumor sections. In total, we included seven biopsy samples for analysis of the STA. The patient was grouped with a control via a microarray, and the other patient presented two histological characteristics (classic and desmoplastic nodular), although by means of immunohistochemistry, we observed GAP1 and YAP1 positivity, which indicated that these samples belonged to the MB-SHH group. We proceeded to perform STA, and fewer patients were positive for MB-WNT, MB-G4 and MB-G3 via microarray gene expression. Our deconvolved analysis focused on the expression profile evidenced by the reference set. For this purpose, we eliminated the expression of sequence tags (ESTs) and redundant markers and selected small profiles by molecular group; these selected profiles consisted of genes that were overexpressed exclusively in the molecular group; our selection included 114 DEGs for WNT, 71 DEGs for SHH, 45 DEGs for G3 and 42 DEGs for G4 (Supplementary Table S3). They were obtained by overlaying the expression profile obtained from MB-WNT, MB-SHH, MB-G3, and MB-G4.

**Figure 5:**
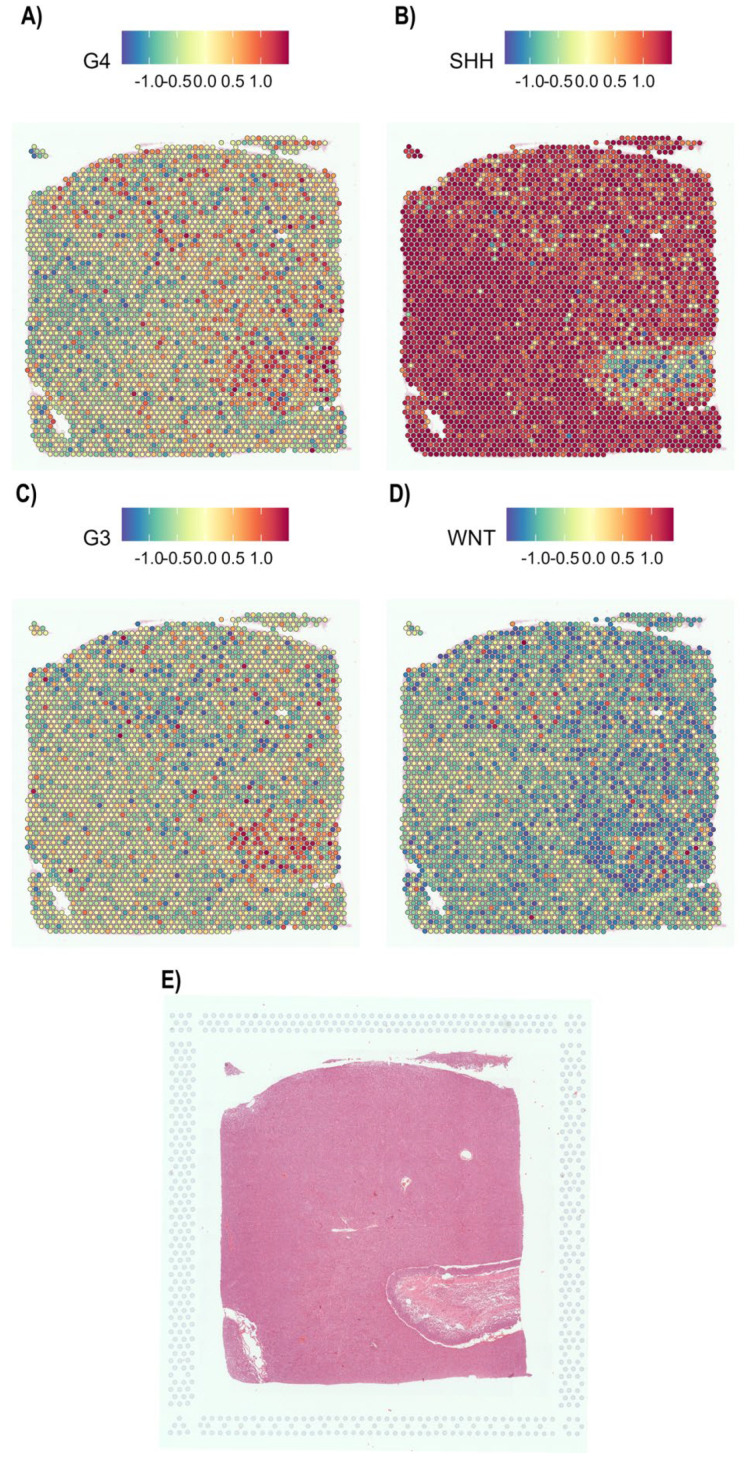
Spatial transcriptomics analysis confirmed the expression profile of the MB−SHHs. The figure shows the deconvoluted signature of the four molecular groups of medulloblastoma. The upper bar indicates the level of expression (blue indicates low expression, and red indicates high expression), and next to the bar, the molecular group is shown. (**A**) Spatial expression profile of MB-G4, (**B**) MB-SHH, (**C**) MB-G3, (**D**) MB-WNT and (**E**) H&E-stained biopsy samples analyzed via STA.

On the basis of our selection, we observed that GEP was associated with four molecular groups of MB. The profile associated with MB-SHH (Figure 5B) is shown in Figure 5, and low-level expression or negative expression was observed for MB-G4 (Figure 5A), MB-G3 (Figure 5C), and MB-WNT (Figure 5D). H&E staining of these biopsies revealed classic histology (Figure 5E). The GEP associated with MB-SHH was replicated in five additional biopsies, as expected (Supplementary Figure S1). Importantly, the biopsies that showed nodular histology presented a heterogeneous profile of MB-SHH-associated expression (Supplementary Figure S1A,C). Unlike what is observed in tissues of the classical lineage (Supplementary Figure S1D,E, Figure 5B).

**Figure 6:**
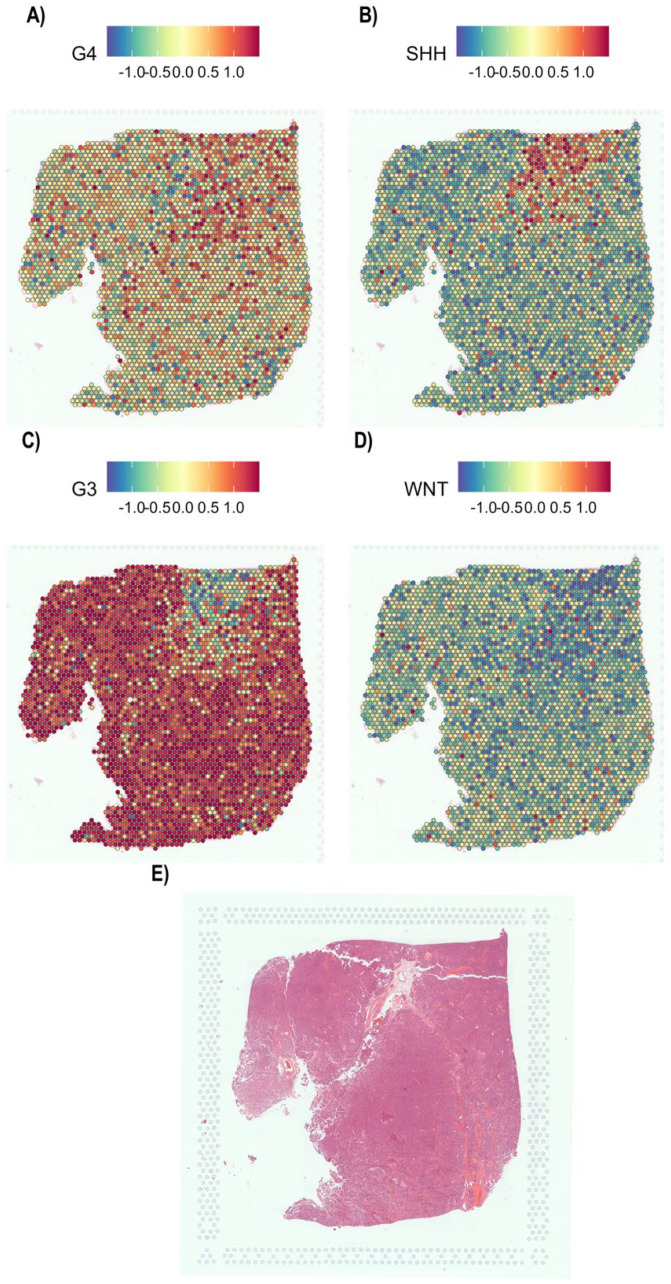
Spatial transcriptomics analysis revealed the expression profile of MB−G3. The figure shows the deconvoluted signature of the four molecular groups of medulloblastoma. (**A**) Spatial expression profile of MB-G4, (**B**) MB-SHH, (**C**) MB-G3, (**D**) MB-WNT and (**E**) H&E-stained biopsy samples analyzed via STA.

On the other hand, in the patient who presented with MB-G3 disease via a microarray, we confirmed the presence of the molecular profile MB-G3 by means of the STA, as expected (Figure 6C). In addition, very low expression associated with MB-G4 was observed (Figure 6A) because of the high similarity of expression between the two molecular groups and the high-level expression associated with MB-G3 (Figure 6C) and the negative profile for MB-WNT (Figure 6D) and MB-SHH (Figure 6B), as expected. H&E staining of these biopsies revealed classic histology (Figure 6E).

**Figure 7:**
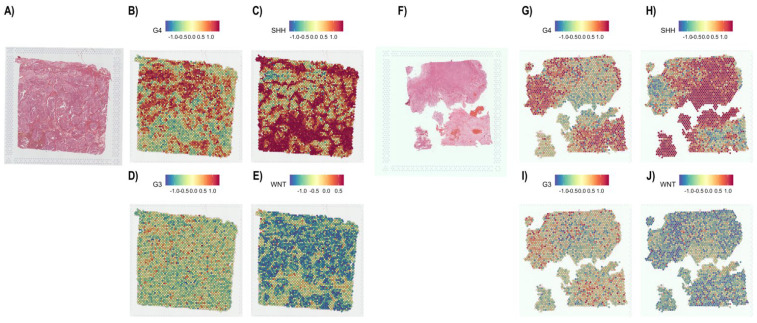
Spatial transcriptomics analysis detected mixed characteristics in patients with medulloblastoma. The figure shows a case of medulloblastoma, represented by two tumor fractions. (**A**–**E**) Fractions of desmoplastic/nodular tumors. (**A**) H&E staining of the biopsy sample analyzed by STA. (**B**) Spatial expression profile of MB-G4, (**C**) MB-SHH, (**D**) MB-G3, and (**E**) MB-WNT. (**F**–**J**) Fractions that exhibited classic tumors. (**F**) H&E staining of the biopsy sample analyzed by STA. (**G**) Spatial expression profiles of MB-G4, (**H**) MB-SHH, (**I**) MB-G3, and (**J**) MB-WNT are shown. Both tumor fractions present an MB-SHH profile in ~80% and ~20% an MB-G4 profile. The positive areas for the MB-SHHs are mutually exclusive for the MB-G4 profile and vice versa.

Finally, we analyzed the patients who presented mixed histological characteristics: desmoplastic/nodular (Figure 7A) and classic MB (Figure 7F). Our findings revealed that these patients exhibited mixed GEP of MB-SHH and MB-G4 in both biopsies by TSA. Approximately 80% of the tissue was MB-SHH (Figure 7C,H), and the remaining 20% had characteristics of MB-G4 (Figure 7B,G). Moreover, low or null expression of MB-G3 (Figure 7D,I) and MB-WNT (Figure 7E,J) was observed. Additionally, regions with MB-SHH expression are mutually exclusive in terms of MB-G4 characteristics and vice versa (Figure 7B,C,G,H). These findings revealed that this patient presented mixed expression characteristics.

## 3. Discussion

Medulloblastoma represents a priority health problem in our country because it is the neoplasm with the highest morbidity and mortality from tumors of the central nervous system. Before the genotype was described, several clinical prognostic factors were used. A complete surgical tumor excision or, if possible, a residual tumor of less than 1.5 sq.cm was performed. Other unfavorable factors are being under 3 years of age, metastasis, and anaplastic histology [3]. Consequently, with the advent of molecular findings, the survival of these children has improved.

To date, the World Health Organization has recognized four molecular groups (WNT, SHH, G3 and G4) [12] and twelve molecular subgroups [17]. Additionally, the molecular classification of MB is the most important prognostic factor [11], since various clinical studies have focused on targeted therapy [13,14,15,18,19], which has promoted survival in these patients to be greater than 85% in developed countries. Unfortunately, in Mexico, there is no molecular evidence of MB, so we do not know the molecular epidemiology of this cancer type, and the most relevant thing is that clinical protocols cannot be initiated; consequently, patients are treated following the technical protocols of cancer treatment in children and adolescents, taking into consideration only the clinical, radiological and histopathological characteristics, which has led to our survival being less than 50% [3].

The molecular classification of medulloblastoma is a great challenge in modern medicine. Although there are several tools for molecular classification, including expression-based platforms such as NanoString and the panel proposed by Kunder et al., as well as mutation-based approaches, these strategies often lack sufficient accuracy for reliable discrimination among the four molecular groups [20,21], and the gold standard remains transcriptomics studies [22]. In low- and middle-income countries, such as ours, alternative classification methods present important practical limitations that restrict their clinical applicability. For example, NanoString-based analysis requires the simultaneous processing of twelve samples, which may delay diagnosis, while mutation profiling and quantitative PCR-based approaches show limited accuracy in distinguishing between groups 3 and 4.

In contrast, our in-silico approach allowed us to overcome one of the principal limitations associated with assignment using expression microarrays: the requirement for large validation cohorts, which, in our context, would mean waiting more than a decade to conduct multicenter studies. However, the increasing availability of publicly accessible microarray datasets [23,24], has enabled the development of alternative analytical strategies [25]. Accordingly, we performed microarray data mining of the four molecular groups of medulloblastoma [26], which included more than 100 confirmed and validated patients with medulloblastoma [10,27,28].

The heterogeneity of medulloblastoma is high, as is the mortality rate in developing countries; however, this tumor can be treated with targeted therapeutic regimens. The molecular groups WNT and SHH do not represent a challenge in the diagnosis because it is possible to identify these groups via immunohistochemistry [16]. However, groups 3 and 4 are very similar molecularly, which makes their diagnosis difficult, and both groups represent ~60% of the cases [29]. On the basis of similarity and dissimilarity and dimensional reduction, we analyzed gene expression profiles via supervised analysis and obtained the molecular landscape of medulloblastoma (Figure 1 and Figure 2). Furthermore, unsupervised analysis via principal component analysis (PCA) was used to predict the molecular groups of patients with medulloblastoma. Although the comparison of data from repositories and Mexican patients with medulloblastoma could reveal a risk of diagnostic bias, the methods of dimensional reduction, which are based on the PCA distance method, could be an excellent tool for the prediction of the molecular medulloblastoma group (Figure 3) [9].

Transcriptomica analysis has led to the precise diagnosis of medulloblastoma [27,30,31]. However, large cohorts of patients must be evaluated to identify molecular patterns, as observed in previous reports from Northcott [32], Kunder [33] and Veiga-Cruzeiro [34]. Unlike these reports, we start from small cohorts or even a single sample. Our PCA-based method could mitigate errors in assignment because extensive numbers of markers are analyzed simultaneously in the microarray (Figure 3). Although the GEA is not suitable for routine diagnostics, we have systematized the process for obtaining fresh tumors during surgery, RNA preservation, purification, and microarray processing, as well as bioinformatics analysis, and the final results are obtained in two weeks; however, the challenge is long-term high cost. Despite this, we have achieved molecular assignment of MB, which is unprecedented for our institution and our country and is important in medical practice today.

The use of immunohistochemical methods for diagnosing medulloblastoma in MB-WNT, MB-SHH and non-MB-WNT/non-SHH tumors has been validated and widely reported [16,35,36]. We confirmed our findings via a microarray. The results obtained by immunohistochemistry were consistent with those reported by Ellison [16]. We observed nuclear positivity of β-catenin for MB-WNT, GAB1 and/or YAP1 for MB-SHH and negativity for MB-G3 and G4 (Figure 4) [16]. Although immunodetection could present biases because of the high degree of tumor heterogeneity and lack of consistent intratumoral groups observed in large cohorts [16], our immunohistochemistry findings validated the prediction of molecular groups via the gene expression profile method, suggesting that it is an adequate method for medulloblastoma molecular assignment and that a low number of samples could be tested to provide a molecular assignment.

Spatial and temporal gene expression is a new discipline for identifying molecular pathogenesis to understand multiple layers of cellular, dimensional and dynamic [37] communication in the context of tumor heterogeneity. It is well known that medulloblastoma has important intra- and intertumor heterogeneity [38,39,40]. However, compared with all the cell transcriptomes, the gene expression patterns associated with the four molecular groups represented a small fraction of the expressed transcripts. Therefore, in clinical practice and for future technological developments focused on molecular diagnosis in MB, the challenge is to develop a panel that allows the differentiation of these groups. For this reason, we performed manual data curation and selection; we obtained a reduced profile for four molecular groups (Supplementary Figure S1, Figure 5, Figure 6 and Figure 7). The exploration of these profiles for diagnostic utility was in accordance with the results of the microarray and immunohistochemistry analyses. These results are very important; in most health care centers, it is easier to handle and preserve fixed-in formol and paraffin-embedded biopsies than to handle fresh biological samples; this approach could be an alternative for the molecular classification of MB, although the cost would be excessive. In addition, long-preserved biopsies also pose a risk owing to high levels of RNA degradation.

Unfortunately, we could not confirm the WNT profile via STA, and the gene expression profiles associated with MB-G3 (Figure 6) and MB-G4 (Figure 7) were limited to two patients, making confirmation of these phenotypes difficult. Childhood biphenotypes are rare and have a poor prognosis because of a mixture of tumor linages [41]; however, in medulloblastoma, no such manifestations have been reported. Our exploration of the molecular architecture of patients who presented with a mixture of classic/desmoplasic tumors revealed two molecular profiles (Figure 7), which suggests that these patients could have mixed tumors. However, there are no reports in the literature in this regard, and unfortunately, there are no molecular studies in the country as a point of comparison. One limitation is that the approaches we performed are expensive. Owing to the lack of molecular diagnoses in our country, several challenges in the development of this project are key to the success of an adequate and conclusive molecular diagnosis of medulloblastoma. These limitations are related to the quality of the RNA used for the microarrays, the quantities of fresh tissue and the adequate preservation of the biopsies embedded in paraffin, which play important roles in the success of the microarrays and the STA assay, respectively.

Despite the above challenges, our study provides relevant information that will promote changes in the management of patients with medulloblastoma in our country, which could lead to improved survival in these patients. However, we still need to delve more deeply into neoplasia since, in the latest WHO update, 12 molecular subgroups of MB have been described, and this classification is based on the genomic and epigenomic expression profile.

## 4. Materials and Methods

### 4.1. Data Mining and Gene Expression Analysis

In this study, we used expression microarray data from the Affymetrix array HG U133_Plus 2 and a dataset that included normal cerebellum, MB-WNT, MB-SHH, MB-G3, and MB-G4 data, which are available from the Gene Expression Omnibus (GEO) (GSE4036, GSE10327, GSE37418, GSE44971 and GSE49243) according to previous reports (Supplementary Table S1) [26]. After that, the gene expression profile (GEP) associated with the four molecular groups of MB was obtained via Partek Genomics Suite v7.18, according to previous reports [26,42,43,44]. In brief, the cerebellum was used as a gene expression baseline and compared against MB-WNT, MB-SHH, MB-G3 and MB-G4. To identify differentially expressed genes (DEGs), we selected those with a fold change (FC) >5 and <−5 with an FDR < 0.005, in accordance with previous studies [26,42,45].

### 4.2. Sample Acquisition

Twenty-five patients with a diagnosis of MB were included (Supplementary Table S2). Informed consent and assent from the patients and their families were signed prior to sample collection. The protocol was approved by the Institutional Ethics Committee (protocol INP 2022/003) CONBIOÉTICA-09-CEI-025-20161215, and the sample was handled in accordance with the Declaration of Helsinki. The tumor tissue was placed in RNAlater (Qiagen, Valencia, CA, USA) and stored at −70 °C until RNA purification.

### 4.3. RNA Purification

The total RNA was purified via a TissueLyser system (Qiagen, Valencia, CA, USA) at 30 Hz for 45 s and subsequently purified via an RNeasy Mini Kit (Qiagen, Valencia, CA, USA) according to previous reports [42,43,46]. The quantification and RNA integrity were evaluated via a NanoDrop One UV–Vis Spectrophotometer (Thermo Fisher Scientific, Waltham, MA, USA) and an Agilent RNA 6000 with an Agilent 2100 Bioanalyzer (Agilent Technologies, Santa Clara, CA, USA), respectively [42]. The purified total RNA was stored at −70 °C until use. The remaining tissue was fixed in formalin and embedded in paraffin for histopathological diagnosis.

### 4.4. Microarray Processing

We used the Affymetrix HG U133_Plus 2.0 array (Affymetrix, Santa Clara, CA, USA), which contains more than one hundred thousand markers covering forty-seven thousand transcripts according to the data sheet. The samples were processed according to the technical specifications for this microarray. Briefly, 100 ng of total RNA was used, and in vitro transcription and cDNA synthesis were carried out, after which the RNA was fragmented and labeled. Hybridization was performed in the hybridization oven at 60 rpm at 45 °C for 17 h, after which the samples were washed with the GeneChip 450 fluid station and finally scanned with a 7G GeneChip scanner according to what was established by the commercial house and previous reports [42].

### 4.5. Integration of the Reference Set and Data of Patients Not Classified Molecularly

The transcriptomic signatures of the four molecular groups of MB were obtained from the Affymetrix HG U133_Plus 2 array (data mining) [26] and included as what is referred to as the reference set, and 25 patients who were diagnosed with medulloblastoma between January 2022 and December 2025 at the Instituto Nacional de Pediatria and the Hospital Infantil de Mexico “Federico Gomez”.

### 4.6. Comprehensive Supervised and Unsupervised Analysis of the Medulloblastoma Transcriptome

The analysis included a reference set (*n* = 110) and 25 MB cases that were obtained from the two institutions. The CEL files obtained from the molecular groups (reference set) and the unknown cases (25 MB) were loaded with Transcriptome Analysis Console (TAC) V4.0.2.15 software (Affymetrix, Santa Clara, CA, USA). In summary, the CEL files were imported with the TAC files, and the CEL files were transformed into CHP files. The molecular group was assigned for each sample of the reference set, and the newly diagnosed patients were labeled “unknown (?)”. Quality controls (hybridization and marking) were verified. The data were normalized via robust multichip analysis (RMA). An unsupervised learning method was applied, reducing the dimensionality of the gene expression with principal component analysis (PCA) for the grouping of the unknown samples.

### 4.7. Immunohistochemistry

Immunodetection was performed in an automated way via the Ventana BenchMark Ultra (Roche Diagnostics, Basel, Switzerland) via the Ultra View detection system. Five-micron-thick sections were cut, deparaffinized, hydrated, and conditioned with CC1 buffer. Endogenous peroxidase activity was blocked with 3% hydrogen peroxide. The samples were incubated with primary antibodies against β-catenin (cat. BD610154 at a 1:500 dilution; BD Bioscience, San Jose, CA, USA), YAP1 (cat. sc-101199 at a 1:300 dilution; Santa Cruz Biotechnology, Santa Cruz, CA, USA) and GAB1 1 (cat. sc-133191 at a 1:300 dilution; Santa Cruz Biotechnology). This mixture was subsequently incubated with the polymerized secondary antibody and with the avidin-biotin-peroxidase complex (Roche, Basel, Switzerland). The peroxidase activity was revealed via the chromogen 3,3′-diamino-benzidine, and finally, the sections were mounted on synthetic resin.

### 4.8. Image Acquisition

Ten biopsies were proposed for spatial transcriptomic analysis, but three were excluded because they were not viable for the methodology due to low RNA quality. A total of seven spatial analyses were performed, including MB-SHH (*n* = 4) and MB-G3 (*n* = 1) analyses. In two cases, two tissue sections were analyzed, and in three cases, a single section was analyzed.

From tissue fixed in formol and embedded in paraffin, 5 µm thick sections were obtained and placed on a Visium 2.0 spatial gene expression slide in capture areas of 6.5 × 6.5 mm (10x Genomics, Pleasanton, CA, USA), and RNA integrity [47] for spatial gene expression was evaluated in brief. The tissues were deparaffinized and stained according to the Deparaffinization and H&E Staining Protocol (CG000409, 10x Genomics) by incubating the slide at 60 °C for 2 h in an oven, then immersed in xylene for 10 min, and then in graded ethanol solutions of 100% to 70%, 3 min each; finally, the slides were washed in Milli–Q water for 20 s. The tissues were covered with 1 mL of hematoxylin and incubated at room temperature for 3 min, the dye was removed, and the slides were washed twice in Milli–Q water for 10 s, manuscript in preparation.

The tissues were deparaffinized, and H&E staining was performed according to the manufacturer’s instructions (CG000409, 10x Genomics). Briefly, the tissues were immersed in bluing buffer at RT for 1 min, and the slides were washed in Milli-Q water for 20 s. The tissues were stained with alcoholic eosin at RT for 1 min and washed in Milli-Q water for 20 s. Coverslipping was performed with 85% glycerol. Image acquisition was performed under a brightfield with an objective Plan-Apochromat 20x/0.8 M27 (Carl Zeiss, Göttingen, Germany) in an Axio Imager Z1 microscope (Carl Zeiss, Jena, Germany), and the samples were photographed with a high-speed color camera with a 0.63x adapter (AxioCam; Carl Zeiss, Jena, Germany). The final photomicrographs were obtained with Zen 3.0 software (Zen Pro. Carl Zeiss, Jena, Germany) by the integration of 220 tiles, each autofocused, into a stitched single image approximately 1600 × 1600 pixels.

### 4.9. RNA-Seq and Analysis of Spatial RNA-Seq Data

The RNA messenger released during permeabilization was bound to capture probes on the slide, where cDNA synthesis was performed. After that, the tissue was removed, and the cDNA attached to the spatial barcodes was transferred to a tube to perform PCR amplification according to 10X protocols. Sequencing adapters were attached to the sample via the kit, and finally, the libraries were purified. Libraries were sequenced on the NextSeq 2000 platform (Illumuna, San Diego, CA, USA).

The BCL files generated by the Illumina sequencer were converted to fastq files via the Spaceranger/2.0 implementation (10x Genomics) with its mkfastq function, which integrates the use of bcl2fastq/2.20. The alignment and counting were generated via the count function with the STAR/2.7.2a aligner. The sequences were compared against the reference human genome (GRCh38). The sequenced data were correlated with the spatial coordinates in the H&E-stained image on the basis of the information from the spatial identifiers. All reads and microsites with no associated tissue were removed; the resulting filtered matrix from SpaceRanger was used for subsequent analysis.

The data were processed via the Seurat/5.1.0 package in R/4.4.0 [48]. Microsites with fewer than 500 transcripts and more than 20% mitochondrial transcripts were filtered out. The various samples were initially corrected via the SCTransform function, and the data were integrated by searching for the 3000 most representative transcripts via the IntegrateData function.

### 4.10. Spatial Transcriptomics Samples

Ten slides were initially considered for processing using the 10X Visium FFPE assay. However, only seven met the required quality criterion of DV200 > 50%, indicating that three samples contained highly fragmented RNA in the FFPE tissue sections. The seven qualifying slides corresponded to five patients; two patients were represented by two slides obtained from different sections of the same tumor. Table 1 summarizes the quality metrics for these samples; all seven slides met the quality requirements for inclusion in the analysis.

### 4.11. Deconvoluted Transcriptomic Signature

The transcriptional signatures of the four molecular groups of medulloblastoma obtained via expression microarrays were used for assignment of the spatial transcriptome via the R package Giotto via the runPAGEEnrich function [49]. We used the normalized expression data for each slide, and using the transcriptional signatures of each molecular group, we computed an association score for each spot in the slide. Therefore, all the slide spots were consistent with the four transcriptional signatures. In addition, deconvolution analysis was performed using gene expression profiles associated with four molecular groups (Supplementary Table S3).

## 5. Conclusions

We characterized the gene expression profiles of molecular groups associated with medulloblastoma. Additionally, gene expression profile analysis of medulloblastoma represents a significant advance in precision medicine; integrating different layers of transcriptomic information allows us to demonstrate underlying molecular changes in the four molecular groups of medulloblastoma.

## Figures and Tables

**Table 1:** Sample description and quality metrics.

Subtype	SHH	SHH	SHH	SHH	SHH/G4	SHH/G4	G3
Patient	1	2	3	3	4	4	5
Slide	1 of 1	1 of 1	1 of 2	2 of 2	1 of 2	2 of 2	1 of 1
Number of tissue spots	3457	2714	3817	3613	2385	3984	3361
Mean reads per spot	84,455	122,771	68,597	99,316	120,599	123,135	91,238
Median genes	6525	7755	5594	7422	6980	8465	5346
UMIs per spot	21,664	36,626	16,108	28,242	23,818	44,302	11,461
Sequencing depth	86,425,000	67,850,000	95,425,000	90,325,000	59,625,000	99,600,000	84,025,000
Q30	96.00%	96.6	96.1	95.40%	96.00%	98.70%	96.4
Alignment rate	98.80%	98.9	98.6	98.50%	98.80%	98.30%	98.8
Fraction of mitochondrial reads	<25%	<25%	<25%	<25%	<25%	<25%	<25%
DV 200	>50%	>50%	>50%	>50%	>50%	>50%	>50%

## Data Availability

Personal patient data are not available for ethical reasons. The microarray CEL files are available in the Gene Expression Omnibus under the accession numbers GSE4036, GSE10327, GSE37418, GSE44971 and GSE49243. The raw spatial transcriptomes are available in the GEO under the accession number GSE306506 (manuscript in preparation).

## Supplementary Materials

The following supporting information can be downloaded at: https://www.mdpi.com/article/10.3390/ijms27135720/s1.

## Abbreviations

TAC	Transcriptome analysis console
YAP1	Yes1-associated Transcriptional Regulator
GAB1	Growth Factor Receptor Bound Protein 2-Associated Protein 1
RNA-seq	Ribonucleic acid sequencing
MB	Medulloblastoma
CNS	Central nervous system
WNT	Wnt Family Member 1
SHH	Sonic hedgehog signaling molecule
MB-WNT	Medulloblastoma WNT pathway is activated
MB-SHH	Medulloblastoma SHH pathway is activated
MB-G3	Medulloblastoma of Group 3
MB-G4	Medulloblastoma of Group 4
TP53	Tumor protein p53
GEP	Gene expression profile
GEA	Gene expression analysis
DEGs	Deregulated expressed genes
RNA	Ribonucleic Acid
RMA	Robust Multichip Analysis
PCA	Principal component analysis
RT	Reverse Transcription
cDNA	Complementary Deoxyribonucleic Acid
PCR	Polymerase Chain Reaction
STA	Spatial transcriptome analysis
ESTs	Expression of Sequence Tags
H&E	Hematoxylin and Eosin
WHO	World Health Organization
