# Supplementary material for: Identification of Markers on the Basis of Transcriptomic Analysis for Molecular Assignment of Medulloblastoma
Source: Int J Mol Sci. 2026 Jun 24;27(13):5720. doi: 10.3390/ijms27135720 (PMC13360944; doi:10.3390/ijms27135720)
Supplement: Supplementary file 1 [file ijms-27-05720-s001.zip › Supplementary Table S2.pdf]

Supplementary Table S2. Clinical characteristics of the cohort of Mexican patients studied.

ID patient	Age (years)	Sex	Histology	IHC group	MA group	ST group
MB507	8	Male	Classic	Non-WNT/ Non-SHH	G4	
MB516	2	Male	Desmoplastic/Nodular	SHH	SHH	SHH/G4
MB518	10	Male	Desmoplastic/Nodular	WNT	WNT	
MB02	14	Female	Classic	WNT	WNT	
MB03	2	Male	Medulloblastoma with myogenic differentiation	SHH	G3	
MB1	6 months	Male	Extensive nodularity	SHH	SHH	SHH
MB2	4	Male	Classic	G3	SHH	
MB3	14	Male	Desmoplastic/Nodular	SHH	SHH	
MB4	15	Male	Classic	Non-WNT/ Non-SHH	G4	
MB5	6	Male	Classic	Non-WNT/ Non-SHH	G3	G3
MB6	9	Male	Classic	SHH	CONTROL	SHH
MB7	8	Female	Classic	Non-WNT/ Non-SHH	G3	
MB8	4	Male	Classic	Non-WNT/ Non-SHH	G3	
MB11	11	Male	Classic	Non-WNT/ Non-SHH	G4	
MB13	6 months	Female	Extensive nodularity	SHH	SHH	
MB14	14	Male	Classic	Non-WNT/ Non-SHH	G4	
MB16	2	Male	Classic	G3	G3	
MB17	8	Female	Classic	WNT	WNT	
MB24	6	Male	Extensive nodularity	SHH	SHH	
MB25	10	Male	Extensive nodularity	SHH	SHH	
MB27	2	Female	Classic	SHH	SHH	
MB28	15	Male	Desmoplastic/Nodular	SHH	SHH	
MB29	7	Male	Classic	WNT	WNT	
MB30	2	Male	Classic	Non-WNT/ Non-SHH	G3	
MB31	3	Male	Desmoplastic/Nodular	SHH	SHH	
MB33	3	Female	Desmoplastic/Nodular	SHH	NA	SHH

NA: Not available.

ICH group: Immunohistochemical classification result

MA group: Microarray expression classification result

ST group: Spatial transcriptome classification result.
