# Supplementary figures and images for: Identification of Markers on the Basis of Transcriptomic Analysis for Molecular Assignment of Medulloblastoma
Source: Int J Mol Sci. 2026 Jun 24;27(13):5720. doi: 10.3390/ijms27135720 (PMC13360944; doi:10.3390/ijms27135720)

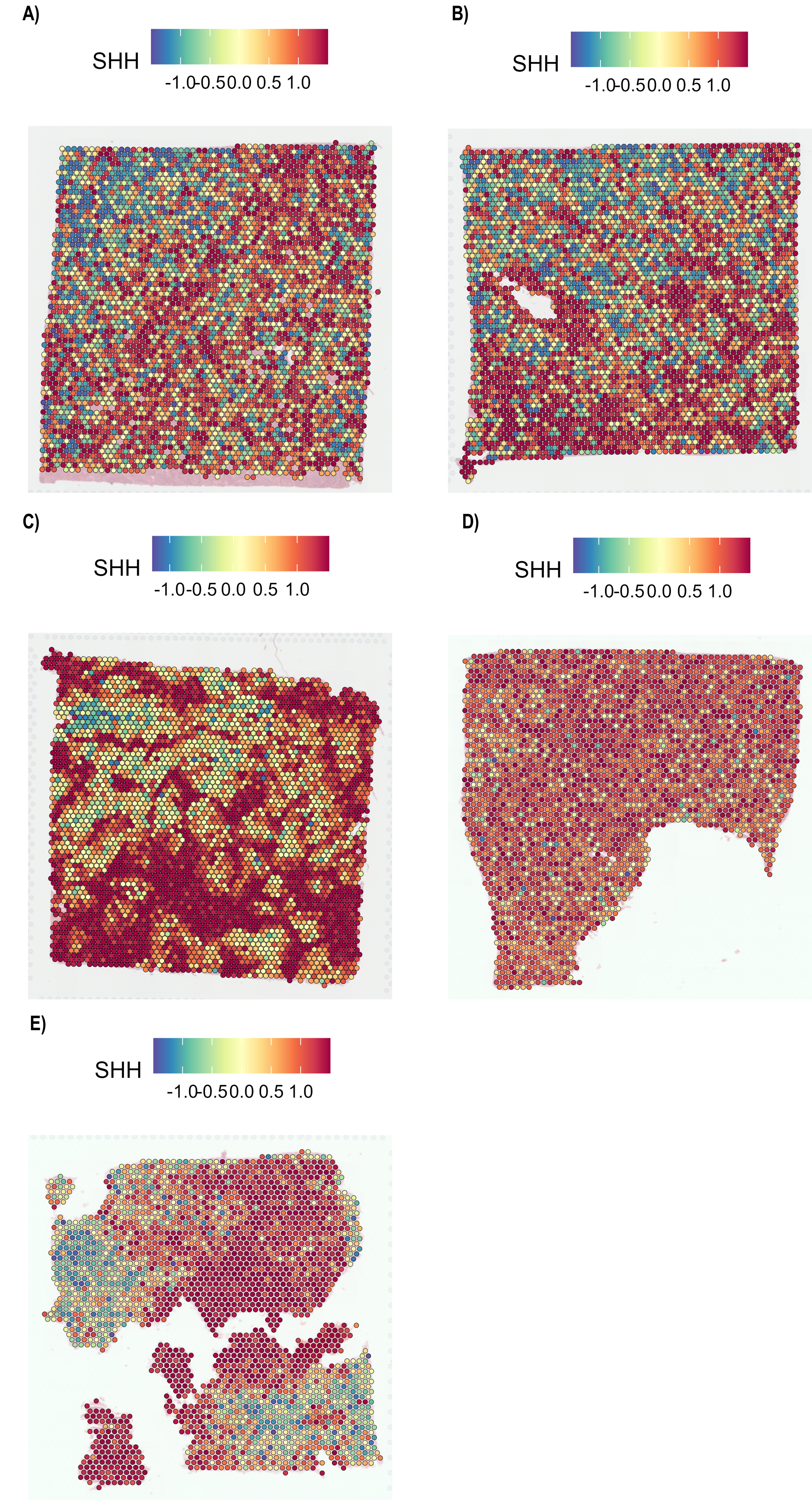

Supplement: Supplementary file 1 [file ijms-27-05720-s001.zip › Supplementary Figure S1.tiff]
